# Feline oral in situ carcinoma associated with papillomavirus infection: A case series of 7 cats

**DOI:** 10.1177/03009858251352594

**Published:** 2025-07-03

**Authors:** John S. Munday, Cynthia M. Bell, Emma L. Gulliver

**Affiliations:** 1 Massey University, Palmerston North, New Zealand; 2 Specialty Oral Pathology for Animals, Geneseo, IL; 3 Laverty Vetnostics, Macquarie Park, NSW, Australia

**Keywords:** Bowenoid in situ carcinoma, cat, FcaPV3, gingival, mucosa, neoplasia, oral, papillomavirus, squamous cell carcinoma

## Abstract

Cutaneous lesions due to papillomavirus (PV) infection are well described in cats. However, there are few reports of similar lesions in the oral cavity. In this case series, 7 cats with in situ carcinomas of the oral mucosa are reported. Lesions appeared histologically like cutaneous Bowenoid in situ carcinomas, and PV-induced cell changes were visible within lesions from 6 cats. A PV etiology was further supported by intense p16^CDKN2A^ protein immunolabeling within all lesions. Five lesions contained Felis catus papillomavirus (FcaPV) type 3 DNA, while 2 contained FcaPV1 DNA. Cats had clinical signs of drooling and oral pain for over 6 months prior to diagnosis, and the dorsal surface of the tongue was most often affected. Four cats had multiple oral lesions, and 2 cats had oral and skin lesions. Of the 6 cats for which clinical outcome was known, 3 are still alive at least 6 months after diagnosis, 2 died of unrelated causes 7 and 14 months after diagnosis, and 1 cat was euthanatized due to oral pain 18 months after diagnosis. Results suggest PV-associated oral in situ carcinoma is a specific disease entity of cats. Lesions are slowly progressive with pain management allowing long survival times. No cases were known to progress to invasive squamous cell carcinoma, and feline oral squamous cell carcinomas appear to infrequently develop as a progression from these lesions. Due to the marked difference in biological behavior, diagnosticians should differentiate PV-associated oral in situ carcinomas and oral squamous cell carcinomas in cats.

Domestic cats are infected by at least 8 different papillomavirus (PV) types.^22^ As in other species, most infections remain asymptomatic; however, PVs are currently associated with both cutaneous and oral lesions in cats.^5,22^ The cutaneous lesions include viral papillomas, viral plaques/Bowenoid in situ carcinomas (BISCs), Merkel cell carcinomas, and a proportion of cutaneous squamous cell carcinomas (SCCs).^8,16,21,26,29^ While multiple different PV types have been reported to cause skin lesions, Felis catus papillomavirus 2 (FcaPV2) and FcaPV3 are most frequently detected.^22,35^ Within the oral cavity, there are rare reports of papillomas due to FcaPV1 and a single report of an in situ carcinoma associated with FcaPV3.^13,17^ In addition, there is some evidence that PVs may cause feline oral SCCs with FcaPV2 DNA detected in 10 of 32 (31%) cases in 1 study and in 12 of 28 (43%) cases in another.^1,37^ However, PV DNA was rarely detected in other similar studies of feline oral SCCs,^4,14,19,23,31,38^ and it is uncertain whether PVs influence the development of these common, often fatal feline cancers.

As PVs are often present asymptomatically, it can be hard to determine the significance of detecting PV DNA in a lesion.^34^ Evidence supporting a causative association includes the presence of PV-induced cell changes within a lesion. However, such changes are rarely present in SCCs, and the absence of cell changes does not exclude a PV etiology. In human pathology, p16^CDKN2A^ protein (p16) immunolabeling is routinely used to detect changes in cell regulation that are consistently caused by PVs during oncogenesis.^32^ A human oral SCC that has intense cytoplasmic and nuclear p16 immunolabeling is considered to have been caused by PV infection. Similarly, in cats, cutaneous BISCs, which are thought to be caused by PV infection, consistently contain intense nuclear and cytoplasmic p16 immunolabeling.^11^ In contrast, this characteristic p16 immunolabeling pattern is rarely observed in feline oral SCCs.^18,23,33^

If feline oral SCCs are caused by PVs, evidence from similar cutaneous lesions suggests some oral SCCs would develop as the result of progression from a PV-induced pre-neoplastic lesion.^12^ Such a pre-neoplastic lesion was recently described in the mouth of a cat. This in situ carcinoma contained FcaPV3 DNA, PV-induced cell changes, and intense p16 immunolabeling.^17^ The purpose of this report is to describe 7 cats that had oral lesions consistent with in situ carcinomas caused by PV infection. As follow-up data were available, how frequently these lesions progress to SCC could be determined. This is the first case series of PV-associated oral in situ carcinomas in cats.

## Materials and Methods

### Case Descriptions

The signalments, disease durations, lesion locations, PV types, and case outcomes for each case are summarized in Table 1.

**Table 1.** Summary data of 7 cats with papillomavirus-associated oral in situ carcinomas.

Case	Breed, sex, and age (years)	Duration of clinical signs (months)	Location of lesions	Associated PV type	Case outcome
1	DSH, MN, 9.5	24	Lower lip	FcaPV3	Unknown
2	DSH, FS, 6.5	9	Multiple caudal hard palate Multiple skin lesions	FcaPV3	Died due to oral pain 18 months after diagnosis
3	DLH, FS, 10	18	Dorsal tongue Ventral tongue	FcaPV1	Alive 12 months after diagnosis
4	DSH, MN, 8.5	11	Dorsal tongue Caudal hard palate	FcaPV3	Alive 13 months after diagnosis
5	Persian, MN, 10	3	Dorsal tongue Lateral edges of tongue Multiple skin lesions	FcaPV1	Died due to unrelated disease 7 months after diagnosis
6	DSH, MN, 13	6	Oropharynx	FcaPV3	Died due to unrelated disease 14 months after diagnosis
7	DSH, MN, 11	24	Dorsal tongue	FcaPV3	Alive 6 months after diagnosis

Cases 1 to 4 were submitted to Specialty Oral Pathology for Animals, USA; case 5 was submitted to Kansas State Veterinary Diagnostic Laboratories, USA; cases 6 and 7 were submitted to Awanui Veterinary Ltd., New Zealand.

Abbreviations: PV, papillomavirus; DLH, domestic longhair; MN, male neutered; FcaPV, Felis catus papillomavirus; DSH, domestic shorthair; FS, female spayed.

#### Case 1

This cat was presented to a board-certified veterinary dentist due to a history of “ulcerated lips” of approximately 2 years (Table 1). Clinical examination revealed a poorly demarcated, 3 cm diameter, irregularly shaped, reddened area on the gingival side of the left lower lip. A single small biopsy from the affected area was taken for histology. The cat was subsequently lost to follow-up.

#### Case 2

**Figure 1.**
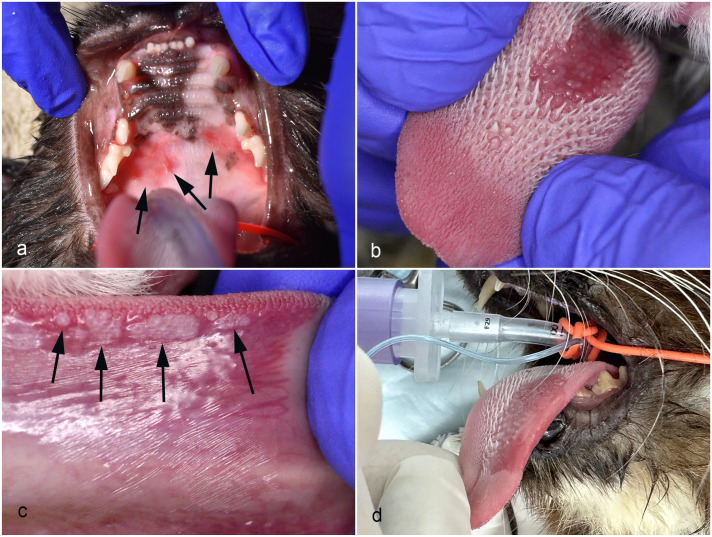
Clinical photographs of cats with papillomavirus-associated oral in situ carcinomas. (a) Case 2. Reddened lesions are visible bilaterally in the mouth caudally on the palate (arrows). (b) Case 5. A well-demarcated reddened area with loss of lingual papillae is visible on the dorsal surface of the tongue. (c) Case 5. Multiple small pale exophytic masses are visible laterally on the ventral surface of the tongue (arrows). (d) Case 7. A well-demarcated area of reddening with loss of lingual papillae is visible on the dorsal surface of the left side of the tongue.

This cat was presented to a board-certified veterinary dentist due to a 9-month history of anorexia and oral pain. Clinical examination revealed reddened, slightly raised plaques bilaterally on the caudal margins of the hard palate (Fig. 1a). The cat also had multiple skin lesions. Samples of a skin mass and the oral lesions were submitted for histology. The cat was treated symptomatically for 18 months before being euthanatized due to increased oral pain and anorexia.

#### Case 3

The cat in this case was initially presented due to clinical signs of oral pain. The cat was treated for stomatitis, and multiple teeth were extracted. However, the cat continued to drool, and reduced grooming was observed. The cat was re-examined 18 months later at a veterinary dentistry specialty practice. A 2 cm × 2 cm area of loss of lingual papillae and reddening was present rostrally on the right dorsal surface of the tongue. In addition, a 0.3-mm diameter distinct nodular mass was visible on the ventral surface of the tongue. Two samples from the dorsal tongue were surgically excised and submitted for histology. The cat was treated symptomatically and remains alive 12 months later, although with reduced grooming.

#### Case 4

This cat was presented to a board-certified veterinary dentist due to ptyalism, decreased grooming, and anorexia of 11 months’ duration. While stomatitis was initially suspected by the referring veterinarian, examination of the mouth revealed reddening and loss of lingual papillae on the dorsal surface of the tongue. A reddened erosive lesion was also visible caudally on the hard palate. Samples of lesions from the tongue and from the hard palate were submitted for histology. The cat was treated symptomatically and examined 13 months later by the same veterinary dentist. The palatal and lingual lesions remained red and erosive with mild progression in the total affected area of mucosa. An additional sample of the lesion was submitted for histology.

#### Case 5

Drooling and halitosis were observed in this cat, and oral examination revealed dental disease as well as a roughly circular area of loss of lingual papillae and reddening centrally on the dorsal surface of the tongue (Fig. 1b). In addition, multiple slightly raised gray plaques were visible ventrally and laterally on the tongue (Fig. 1c). The lesions were still present 3 months later when the cat was clipped, and multiple darkly pigmented skin lesions were also detected. At this time, samples were taken from the dorsal tongue, lateral tongue, and skin and submitted for histology. Four months later, 2 more skin masses were surgically excised as they had become ulcerated and were bleeding. At this time, the tongue lesion was noted to still be present but not to have increased in size or become ulcerated. Three months later, the cat developed hematuria, and a bladder mass was palpated on clinical examination. Urothelial neoplasia was suspected, and the cat was euthanatized with no necropsy performed.

#### Case 6

This cat initially developed ulcerative lesions on the nasal planum, which were treated with cryotherapy without any diagnostic testing. Two months later, the cat developed signs of oral pain and examination revealed a reddened, slightly raised 3 cm diameter area within the oropharynx. The oral lesion had not resolved 6 months later, and 2 samples of the affected area were submitted for histology. Approximately 1 month after the samples were taken, the cat stopped showing clinical signs of oral pain, and an examination of the oral cavity revealed that the lesions appeared less reddened. Fourteen months later, the cat developed skin lesions that were histologically diagnosed as cutaneous hemangiosarcoma. Metastases were detected, and the cat was euthanatized. No necropsy was performed, but examination of the mouth revealed that the oral lesion was still present, although it had not progressed since the time of diagnosis.

#### Case 7

A lesion was noticed on the dorsal surface of the tongue during a routine dental health check. This lesion was initially treated with antibiotics; however, the lesion rapidly increased in size over the following 2 months. At this time, the lesion was a well-demarcated, 2 cm diameter, reddened area with loss of lingual papillae rostrally on the left side of the dorsal surface of the tongue (Fig. 1d). The cat was noted to be drooling and grooming less frequently. The lesion was treated symptomatically for 24 months during which time the lesions neither progressed nor resolved. At this time, the cat was presented at a veterinary dentistry specialty practice, and a sample was taken from the reddened area for histology. The cat is alive 6 months after diagnosis. She has no anorexia but drools blood-tinged saliva and is reluctant to groom.

### Papillomavirus Amplification and p16 Immunohistochemistry

DNA was extracted from formalin-fixed paraffin-embedded tissue scrolls from samples of the oral lesions from each of the 7 cats. All DNA was extracted using a NucleoSpin DNA FFPE XS kit (Macherey-Nagel GmbH, Duren, Germany) according to the manufacturer’s instructions. The consensus primers MY09/11 and CP4/5 were used as previously described.^25^ DNA extracted from a cutaneous viral plaque that contained FcaPV3 DNA was used as a positive control for both primers, while no template DNA was added to the negative control reactions. In addition, specific primers^20^ were used to detect FcaPV2 DNA within the samples using the same reaction conditions as the consensus primers. DNA extracted from a feline cutaneous SCC that contained amplifiable FcaPV2 DNA was used as the positive control, while no template DNA was added to the negative control. DNA amplified by the consensus primers was purified and sequenced as previously described.^25^ The DNA sequences were then compared to known DNA sequences using the BLAST tool (https://blast.ncbi.nlm.nih.gov/Blast.cgi).

Immunohistochemistry for p16 was performed as previously described.^15^ Briefly, sections were heated in a pressure cooker for 10 minutes in 0.01M citrate buffer pH 6.0 with a 10-minute cool down. Endogenous peroxidases and non-specific labeling were blocked before slides were incubated for 60 minutes with a mouse anti-human p16 monoclonal antibody (BD Biosciences, San Jose, California, clone G175-405) at a dilution of 1:50. The antibodies were visualized using a biotinylated horse-anti-mouse/rabbit secondary antibody, a biotin-avidin complex, and 3,3-diaminobenzidine substrate (Liquid DAB Substrate Chromagen System, Dako, Carpinteria, California). A section of cutaneous viral plaque known to contain FcaPV3 DNA was used as the positive control. No primary antibody was used for the negative controls. In addition, as no internal negative control was present in some samples (due to a lack of normal-appearing epithelium), a sample of thickened feline oral mucosa that did not contain amplifiable PV DNA or PV-induced cell changes was also immunolabeled.

## Results

### Histology

**Figure 2.**
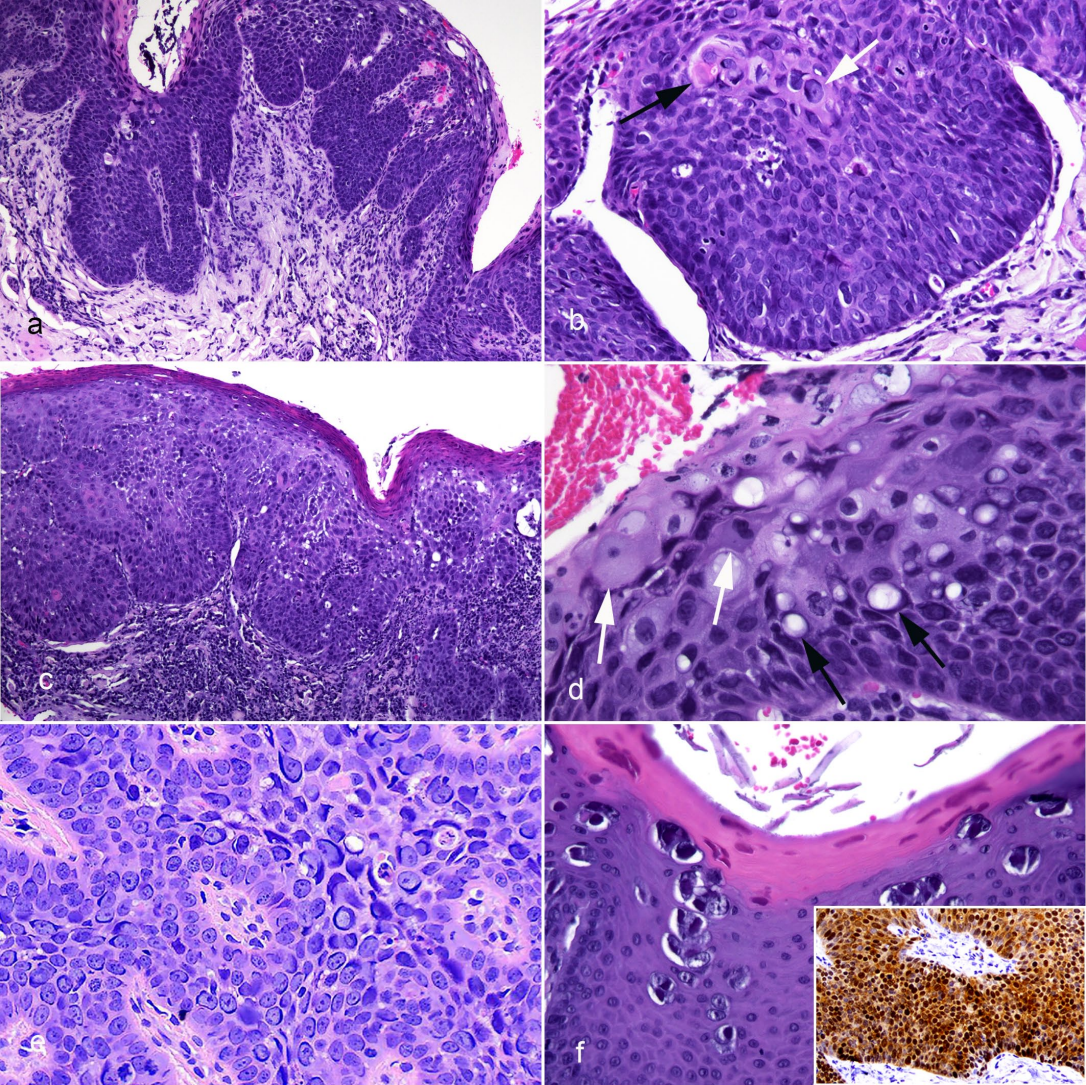
Photomicrographs of papillomavirus-associated oral in situ carcinomas in cats. Hematoxylin and eosin. (a) Case 4, dorsal tongue. The epithelium is irregularly thickened with rounded trabeculae extending into the underlying lamina propria. There is no infiltration through the basement membrane. (b) Case 1, lower lip. As is seen in cutaneous Bowenoid in situ carcinomas, the basal cells are crowded and vertically elongated. Keratinized cells are present within deeper layers of the epithelium (black arrow), and cells with basophilic elongated perinuclear cytoplasmic bodies are visible (white arrow). (c) Case 3, dorsal tongue. The thickened epithelium lacks regular organization, and a prominent layer of lymphocytic inflammation forms a band within the underlying lamina propria. (d) Case 4, dorsal tongue. Clear vacuoles are prominent within the epithelial cells (black arrows). In addition, cells containing increased quantities of blue-gray cytoplasm are visible superficially within the thickened epithelium (white arrows). (e) Case 4, palate. Numerous elongated basophilic cytoplasmic bodies are visible surrounding the nuclei within the affected epithelium. Felis catus papillomavirus (FcaPV) 3 DNA was amplified from this lesion. (f) Case 5, lateral tongue margin. The sample contains large brick-like cytoplasmic bodies that are surrounded by a clear halo. Compared to the other lesions, the thickening of the epithelium appears more orderly with little dysplasia. This lesion contains FcaPV1 DNA. Inset. Case 3, dorsal tongue. Epithelial cells show intense diffuse nuclear and cytoplasmic p16^CDKN2A^ protein immunolabeling. p16^CDKN2A^ immunohistochemistry.

Lesions from all 7 cats appeared as irregular thickening of the epithelium, with the thickened epithelium forming rounded trabeculae that bulged into the underlying lamina propria without invading the basement membrane (Fig. 2a). Cells within the basal layers were crowded and vertically elongated, resulting in a “windblown” appearance (Fig. 2b). Keratinized cells were visible within the hyperplastic epithelium, suggesting loss of normal maturation. In addition, cells within superficial layers of the epithelium in most lesions were keratinized but had dark prominent nuclei. Cases 1, 6, and 7 had the least dysplasia with only mild loss of normal orderly arrangement and maturation of the epithelial cells. Lesions from these cases had only mild inflammation with small numbers of lymphocytes and plasma cells visible within the lamina propria. Inflammation was more marked in cases 2 and 3, with large numbers of lymphocytes and plasma cells forming a distinct narrow band underlying the thickened epithelium. More marked dysplasia was visible within cases 2, 3, 4, and 5. This appeared as greater crowding and disorganization of cells deep within the epithelium, more irregular thickening of the epithelium, and an increased number of keratinized cells within deeper layers of the thickened epithelium (Fig. 2c). Cells within the superficial layers of the epithelium in cases 4 and 5 contained large clear vacuoles. It could not be determined if these vacuoles were within the nuclei or adjacent to, but distorting, the cell nuclei (Fig. 2d). Small foci of ulceration were visible in cases 3 and 5. Features interpreted as PV-induced cell changes were visible in all cases except case 3. Within cases 1, 2, 4, 6, and 7, 30% to 50% of cells contained basophilic cytoplasmic bodies that most often were slender and elongated and partially surrounding nuclei (Fig. 2e). Less frequently, the basophilic material formed brick-like cytoplasmic bodies. In addition, small numbers of cells containing markedly increased blue-gray, slightly granular cytoplasm were visible. Cytoplasmic bodies were also visible in the sample taken from the lateral margin of the tongue in case 5. These bodies were darker, larger, and almost always surrounded by a clear halo (Fig. 2f). In contrast, no PV-induced cell changes were visible in samples of more dysplastic epithelium from case 5. None of the lesions were completely excised, with the epithelial thickening and dysplasia extending to the edges of the tissue margins in all cases. When re-sampled 13 months after diagnosis, the palatal lesion from case 4 was nearly identical to the original biopsy sample; perinuclear basophilic cytoplasmic bodies were readily identified.

Examination of the samples of skin lesions from cases 2 and 5 revealed epithelial thickening and dysplasia with both lesions classified as cutaneous BISCs.

### Polymerase Chain Reaction and p16 Immunohistochemistry

Papillomaviral DNA was amplified from samples from all 7 cats using the CP4/5 primers. Sequencing revealed that the oral lesions from cases 3 and 5 contained DNA sequences nearly identical to FcaPV1 DNA (GenBank number AF480454), while the oral lesions from cases 1, 2, 4, 6, and 7 contained sequences nearly identical to FcaPV3 DNA (GenBank number JX972168). Differences between the amplified sequences and sequences in GenBank were considered to be due to errors in the sequencing process rather than evidence of different PV types. The skin lesion from case 5 contained FcaPV1 DNA sequences, while the skin lesion from case 2 was not available for testing. The MY09/11 primers amplified PV DNA from cases 1, 2, 4, 6, and 7. Sequencing revealed the presence of FcaPV3 DNA. As the MY09/11 primers are known to not amplify FcaPV1 DNA, no PV was amplified from either case 3 or 5 by this primer set. Results from both positive and negative controls were as expected. The primers specific for FcaPV2 amplified DNA from the positive control, but not from any of the oral lesions or the negative control.

Intense cytoplasmic and nuclear p16 immunolabeling was present within the oral lesions from all 7 cats (Fig. 2f, inset). In case 5, sections from both the central ulcerated area and the lateral tongue contained prominent p16 immunolabeling. Examination of the sample of oral epithelium that did not contain evidence of PV infection revealed only patchy p16 immunolabeling that was restricted to the nuclei of basal cells.

## Discussion

This is the first series of in situ carcinomas from the oral cavity of cats. The lesions all appeared similar histologically as well as sharing histological features with cutaneous BISCs. Histological features present in both the oral lesions and cutaneous BISCs included irregular epithelial thickening with variably sized rounded trabeculae that protrude into the underlying lamina propria and vertical elongation of cells in the basal layer of the epithelium.^27^ In addition, all except 1 oral in situ carcinoma contained cell changes caused by PV infection, a feature that is also often present in cutaneous BISCs.

Papillomavirus DNA was amplified from all 7 oral in situ carcinomas. As most PV infections are asymptomatic, detecting PV DNA within a lesion does not indicate causality.^34^ However, in the present cases, a causative role of the PV is supported by the histological similarity of the oral lesions to cutaneous BISCs, which are thought to be caused by PV infection. In addition, the presence of PV-induced cell changes within the lesions indicates the neoplastic cells were infected by the PVs, and the PVs were influencing normal cell regulation. Finally, all 7 oral in situ carcinomas contained intense p16 immunolabeling. Similar immunolabeling has been reported in feline cutaneous BISCs and human PV-induced oral neoplasms.^10,15^

The oral lesions in 5 of the cats were associated with FcaPV3 with the remaining 2 associated with FcaPV1. When lesions containing the 2 different PVs were compared, only lesions associated with FcaPV1 had areas in which cell proliferation was so marked that a distinct mass could be identified. In addition, only the lesions that contained FcaPV1 contained areas of ulceration. Cytoplasmic bodies were visible in all lesions except 2 in situ carcinomas associated with FcaPV1. FcaPV3 has been previously reported in an oral in situ carcinoma and is frequently detected in cutaneous BISCs and SCCs.^17,28,38^ FcaPV1 has previously been reported as the likely cause of feline oral papillomas.^13^ While the areas on the lateral tongue of case 5 contained only mild dysplasia, the diffuse thickening and moderate dysplasia seen in the dorsal tongue lesion from this cat and from samples from case 3 prompted a diagnosis of in situ carcinoma. These results suggest FcaPV1 may cause both hyperplastic papillomas and in situ carcinoma in the mouth of cats.

While there is still uncertainty regarding the role of PVs in feline oral SCCs, FcaPV2 DNA was detected in between 7% and 40% of these cancers in 3 previous studies.^1,2,37^ In contrast, FcaPV2 was not amplified using specific polymerase chain reaction (PCR) primers from any of the oral in situ carcinomas in the present study. A lack of association with FcaPV2 was also supported in 6 of the cases by the presence of PV-induced cell changes that were consistent with those described for FcaPV3 or FcaPV1 but not with those described for FcaPV2.^27^ However, a more general role of FcaPV2 in the development of feline oral cancer cannot be excluded, as the cases in the present study were selected due to the presence of histological features of PV infection. Therefore, it remains possible FcaPV2 could cause oral neoplasia, but these neoplasms do not develop as progression from an in situ carcinoma that contains histological features of PV infection. In addition to being previously detected in a feline oral in situ carcinoma, FcaPV3 was also detected in 5% of feline oral SCCs in 2 studies, although neither histological nor immunohistochemical features were reported.^2,4^ Therefore, while FcaPV3 appears to be the most common cause of oral in situ carcinomas in cats, the role, if any, of this PV in the development of oral SCCs remains uncertain.

The lesions in the present series most often involved the dorsal surface of the tongue and palate with the lips and oropharynx affected less frequently. This distribution is different from feline oral SCCs, in which the gingiva and sublingual region are most often affected.^3,24^ The different lesion distribution adds evidence that oral SCCs infrequently develop as a progression of a PV-associated in situ carcinoma. Most of the cats with oral in situ carcinomas presented due to oral pain, ptyalism (drooling), decreased grooming, or reduced appetite, which are all clinical signs commonly seen with oral SCCs.^3^ However, the cats with in situ carcinomas had clinical histories of months or years, which would be unexpected in cases of oral SCCs.^3^ In addition, 4 of the 7 cats had multiple oral lesions. As multiple oral SCCs are extremely rare in cats,^3^ detecting multiple oral lesions may be useful to help differentiate a PV-associated in situ carcinoma. The possibility of PV-induced oral lesions should also be considered when both oral and skin lesions are present, as was seen in 2 of the 7 cases in this series.

Of the 6 cats for which the outcome was known, only 1 cat was euthanatized due to the oral lesions and the cat survived 18 months after diagnosis. Two cats died of unrelated causes, 7 and 14 months after diagnosis. Three cats remain alive with 2 of these cats surviving over a year from diagnosis without significant worsening of clinical signs. Therefore, while additional cases are required, PV-associated in situ carcinomas appear to progress slowly, and symptomatic therapy (predominantly pain management) may allow long survival times after diagnosis. This information is important to avoid aggressive surgical treatment that, especially in cats, is associated with a significant risk of intractable anorexia.^30^ The slow progression contrasts the aggressive clinical behavior of feline oral SCCs, which are highly infiltrative, resulting in median survival times in the range of 4 to 8 weeks.^7,9^ The marked difference in survival times between the PV-associated oral in situ carcinoma and the more common feline oral SCC suggests diagnostic pathologists should clearly differentiate the 2 entities when examining samples from the oral cavity. An area of dysplasia within the oral epithelium of a cat should not be assumed to be an oral SCC. Oral epithelial dysplasia with PV-induced cell changes or intense p16 immunolabeling is consistent with a diagnosis of PV-associated oral in situ carcinoma and should not be presumed to be a precursor of invasive SCC. Pathologists should refrain from suggesting such an eventuality in comments provided.

Why the 7 cats developed these lesions is uncertain. Papillomaviruses are thought to commonly asymptomatically infect the skin of cats and cutaneous BISCs are thought to develop due to an inability of the immune system to prevent viral replication.^22^ Considering the similar histological appearance of the oral and cutaneous lesions, it may be most likely that the development of oral in situ carcinomas is also strongly influenced by the host immune system. A host immune dysfunction is also supported by the presence of multiple lesions either within the mouth or involving the mouth and skin in 4 of the 7 affected cats. As is the case with most cutaneous BISC cases,^36^ none of the cats had obvious signs of immunosuppression or a history of immunosuppressive therapy. Unless an underlying immunodeficiency can be identified and resolved, there are no proven medical treatments. Imiquimod cream has been used to treat cutaneous BISCs, but toxicity would prevent this treatment for oral lesions.^6^ There is currently no evidence that antiviral or immune-stimulating therapies are beneficial.

The development of 7 cases showing similar histology and clinical behaviors suggests that PV-associated oral in situ carcinomas are a distinct entity in veterinary medicine. Such lesions appear rare but should be suspected when examining long-standing or multiple oral lesions, especially those on the dorsal tongue. Histology reveals features of cutaneous BISCs, and PV-induced cell changes are often visible. The lesions appear slowly progressive and symptomatic therapy may allow long survival times.
